# Measuring Social Contacts in the Emergency Department

**DOI:** 10.1371/journal.pone.0070854

**Published:** 2013-08-21

**Authors:** Douglas W. Lowery-North, Vicki Stover Hertzberg, Lisa Elon, George Cotsonis, Sarah A. Hilton, Christopher F. Vaughns, Eric Hill, Alok Shrestha, Alexandria Jo, Nathan Adams

**Affiliations:** 1 Department of Emergency Medicine, Emory University, Atlanta, Georgia, United States of America; 2 Department of Biostatistics and Bioinformatics, Emory University, Atlanta, Georgia, United States of America; Institut Pluridisciplinaire Hubert Curien, France

## Abstract

**Background:**

Infectious individuals in an emergency department (ED) bring substantial risks of cross infection. Data about the complex social and spatial structure of interpersonal contacts in the ED will aid construction of biologically plausible transmission risk models that can guide cross infection control.

**Methods and Findings:**

We sought to determine the number and duration of contacts among patients and staff in a large, busy ED. This prospective study was conducted between 1 July 2009 and 30 June 2010. Two 12-hour shifts per week were randomly selected for study. The study was conducted in the ED of an urban hospital. There were 81 shifts in the planned random sample of 104 (78%) with usable contact data, during which there were 9183 patient encounters. Of these, 6062 (66%) were approached to participate, of which 4732 (78%) agreed. Over the course of the year, 88 staff members participated (84%). A radiofrequency identification (RFID) system was installed and the ED divided into 89 distinct zones structured so copresence of two individuals in any zone implied a very high probability of contact <1 meter apart in space. During study observation periods, patients and staff were given RFID tags to wear. Contact events were recorded. These were further broken down with respect to the nature of the contacts, i.e., patient with patient, patient with staff, and staff with staff. 293,171 contact events were recorded, with a median of 22 contact events and 9 contacts with distinct individuals per participant per shift. Staff-staff interactions were more numerous and longer than patient-patient or patient-staff interactions.

**Conclusions:**

We used RFID to quantify contacts between patients and staff in a busy ED. These results are useful for studies of the spread of infections. By understanding contact patterns most important in potential transmission, more effective prevention strategies may be implemented.

## Introduction

### Background and Rationale

Presentation of an infectious patient to an emergency department (ED) brings a substantial risk of cross infection. ED cross infection risk was demonstrated dramatically during the 2003 severe acute respiratory syndrome coronavirus (SARS Co-V) epidemic. The son of the first index case arriving in Toronto fell ill after caring for his mother [1]. He visited a crowded ED and waited hours for a hospital bed assignment. Subsequently, 126 nosocomial SARS infections among patients and staff were traced to direct or indirect exposure to this patient; several of these victims died. Since this incident, ED crowding has worsened, [2] increasing commingling of acutely infected patients with other susceptible and high-risk patients, thereby increasing the risk of ED cross infection. This SARS outbreak is not an isolated incident. ED visits have previously been shown to be a significant risk factor for subsequent infection in the pediatric population, [3], [4] as well as in the elderly [5]. Cross infection of patients in the ED is also an important concern to patients and staff in other hospital areas, since more than 40% of hospitalized patients originate from the ED [6].

Annually, a global epidemic of influenza results in significant morbidity and mortality [7], [8]. Although evidence suggests influenza may be transmitted via airborne and contact routes, [9] most authorities agree influenza is transmitted primarily in droplets passing between people, as in the SARS Co-V outbreak [10]. Droplet-mediated cross infection typically occurs within a one (1) meter (m) radius between source and exposed, as the droplets are of adequate size that gravity pulls them to the ground before they can travel laterally [9].

Until recently, many mathematical models of the cross infection process had assumed that individuals are mixing and coming into contact with each other randomly [11]. Recent research has begun to show that humans do not come into contact with each other according to a uniformly random process; human interaction is highly influenced by other external factors [12]–[15]. Knowledge of the social and spatial structure of interpersonal contacts in the ED will provide information useful for building biologically plausible mathematical models of cross infection risk, [16] which can guide development of cross infection control measures.

Advances in technology have made the automated tracking of individuals possible and increasingly affordable using a variety of types of real time location sensing systems, such as radiofrequency identification (RFID) and motes. While the manufacturing and retail sectors of our economy have been making use of such technology to track goods for years, declining costs have enabled researchers to deploy such systems to investigate human motion in a variety of settings. Although investigations of human movements and resulting contacts have been conducted in a variety of settings, such as schools [17], [18] and academic conferences, [14], [19] there has been relatively limited deployment for research purposes in the health care setting. Notably there have been four such studies in settings such as a pediatric emergency department, [20] two hospital wards with airborne precautions, [21] a general pediatric ward, [22] and a medical intensive care unit (MICU) [23]. We report here the results of a year-long deployment of a RFID system covering all areas of an adult ED, describing the contacts between and among patients and staff.

### Objective

The goal of this study was to determine contact characteristics among patients and staff in the ED of a busy urban hospital. The number and duration of contacts between individuals is described overall and by patient-patient (PP), patient-staff (PS), and staff-staff (SS) type contacts.

### Methods

Ethics Statement: The Emory University Institutional Review Board (IRB) granted waiver of all elements of informed consent and waiver of HIPAA authorization.

### Study Design

This is a prospective study.

### Setting

The study was conducted in the ED of Emory University Hospital Midtown in the midtown area of Atlanta, GA. The ED occupies 25,000 square feet, and includes triage, fast track, acute care, and observation functions. It has an annual census of 57,000 visits.

### Contacts

**Figure 1:**
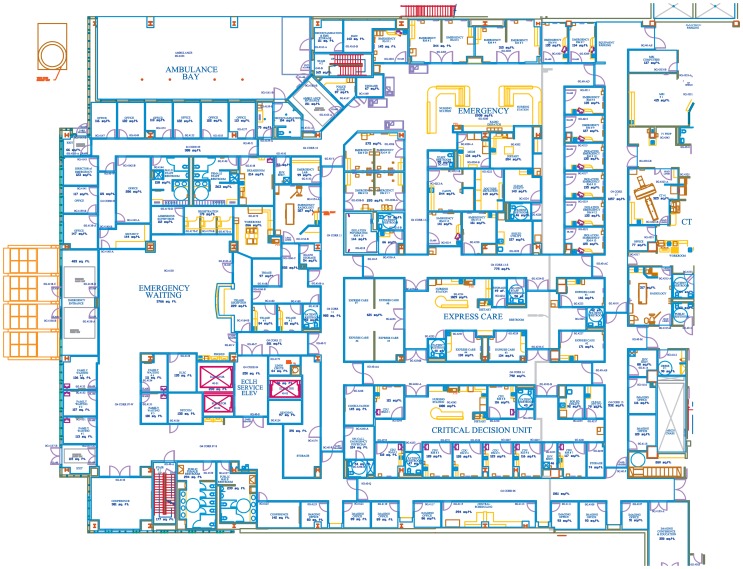
Floor plan of ED at EUHM. Red dots indicate RFID sensors. Zones are numbered and outlined in blue.

Contact was defined as any two individuals located within 1 m of each other in contiguous two-dimensional space, measured by radiofrequency identification (RFID). An active RFID proximity detection system was installed in 2008 and activated in early 2009 (Radianse Corp., Amherst, MA). The ED was divided into 89 two-dimensional zones, and these zones were configured around hard and soft architectural features such that when two individuals were in the same zone simultaneously, they were within 1 m of one another with a very high probability. The floor plan of the ED as divided into zones is given in Figure 1. All areas of the ED were covered by the system.

RFID tags transmitted their unique identifier every ten seconds. The system was dispersed such that RFID tag signals would be detected by at least three receivers. Receivers relayed information back to a server, where a proprietary algorithm determined tag location and assigned a corresponding zone identifier. To facilitate line-of-sight determination in the presence of many walls (i.e., two subjects on either side of a wall but otherwise within 1 m would not be considered as in contact), the ED was divided into zones. Data were retrieved using MySQL Database (Oracle Corp., Redwood Shores, CA) queries and stored in Microsoft Excel (Microsoft Corp., Redmond, WA).

### Time Period Selection

A representative sample of 104 12-hours shifts over one year were randomly selected for direct observation. Two 12-hour periods (one 7am–7pm (day shift) and one 7pm–7am (night shift)) were randomly selected for study from each week between July 1 2009 and June 30 2010. This selection was made in order to get a sense of seasonal variability and to ensure that day and night shifts were equally represented throughout the year. Week was chosen as a blocking factor for the design since there is a distinct rhythm to patient- and work-flow in the ED within a week. The study budget was designed to support the study of no more than two shifts per week.

### Participants

#### Staff

RFID tags were issued to assenting ED staff members to wear.

#### Patients

Immediately prior to each study period, the research team placed RFID tags on assenting patients already enrolled in the process of care in the ED. Research team members placed tags on newly arriving assenting patients during the study period. Psychiatric patients, patients in police custody, and patients who were expected to be discharged within a short time were not approached for RFID tagging, due to either IRB concerns (first two groups) or study staff limitations (last group). Patients in this last group were generally waiting in an exam room and had very few contacts. ED staff removed patient tags at the earliest of time of patient discharge (for those discharged), time of hospital admission (for those admitted), or time of study period conclusion.

### Variables

Data were collected from three different sources. The electronic health record (EHR) (Cerner Millennium Electronic Health Record, Cerner Corp., Kansas City, MO) provided standard clinical and demographic characteristics for patients. The tag identification database tracked tag information. The Radianse database provided time-stamped information about tag zones.

Race was abstracted from the EHR as entered by hospital registration staff, who as part of routine operations ask patients to identify their race and ethnicity. This variable is reported since social mixing may vary culturally and thus it may impact the ultimate generalizability of our study.

**Figure 2:**
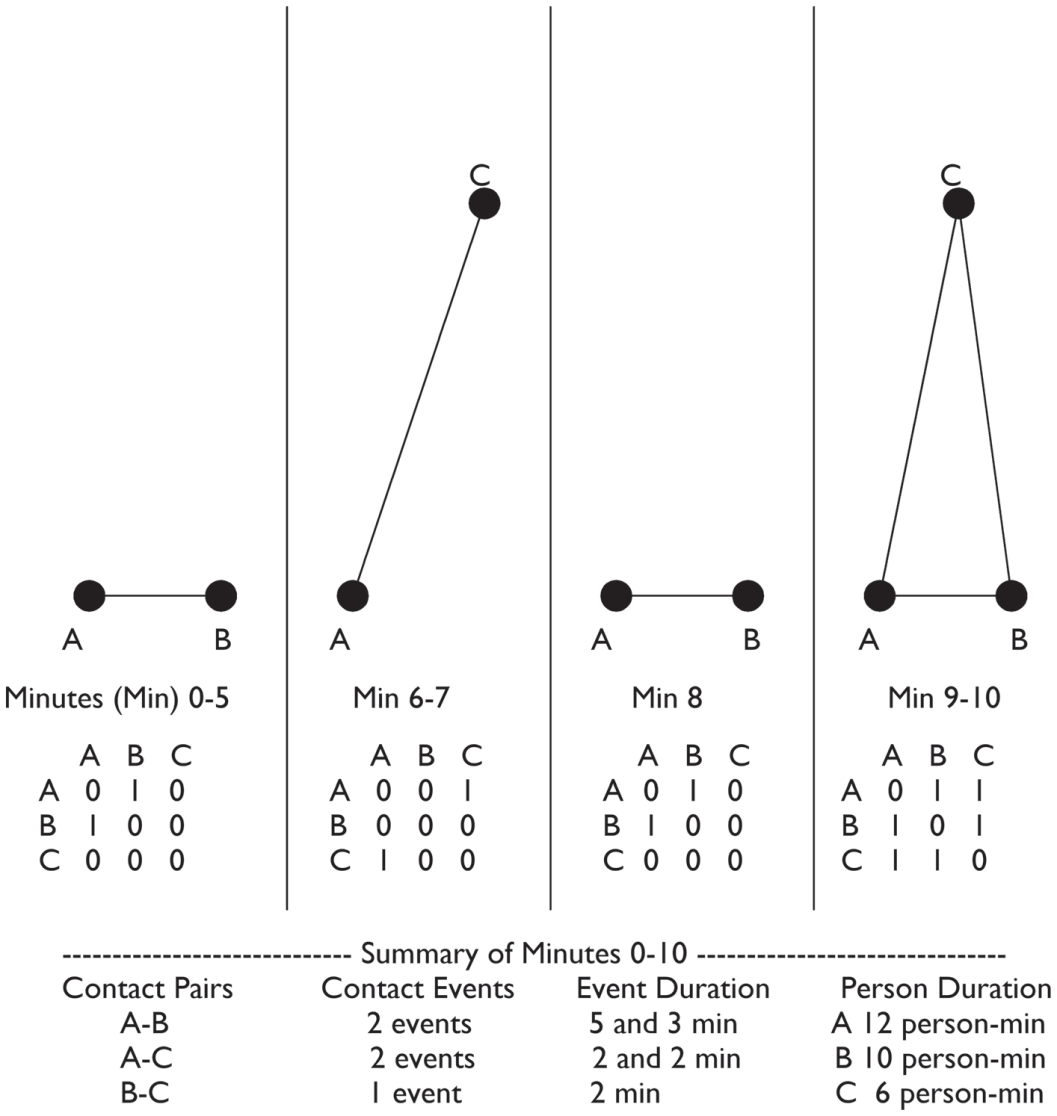
A schematic representation of contact, event, and duration. The schematic above demonstrates how three individuals (A, B, and C) come into contact with each other over a 10-minute period.

Contacts were described in 2 ways. A unique pair of individuals could make contact multiple times over the course of a shift. “Contact pairs” defined multiple contacts as one pair and the duration of these contacts were cumulated. In contrast, “discrete contact events” treated multiple discontinuous instances as multiple contacts of one contact pair. Figure 2 gives a schematic representation of these definitions.

For each second of each shift we created adjacency matrices in which every participant in that shift was cross-listed against every other participant. By aligning these matrices along the dimension of time, we created a 3-dimensional time-resolved adjacency object (TRAO) for each shift in the study. We used data from the Radianse system to determine if any two participants were in contact at a given instance, assigning 1′s in the appropriate cells of the relevant adjacency matrix for that instance. Otherwise 0′s were assigned if participants were not in contact at that time or if neither participant was present in the ED at that time. By summing in the time dimension over these objects we could determine the number of contact pairs formed among participants and the total duration of contact for each pair. We then examined the contact sequence for each contact pair in the time dimension as represented by a sequence of 1′s and 0′s. Each distinct sequence of 1′s that was interrupted by 0′s was called a contact event. This is also represented in Figure 2.

### Efforts to Minimize Bias

#### Selection bias

To reduce selection bias, study periods were randomly selected. We aimed to study every assenting patient and ED-based staff member present in the ED during these periods.

#### Measurement bias

To reduce measurement bias, ED staff were advised to utilize standard operating procedures when performing data entry into the clinical information system. No subjects were asked to record study-specific information. The research team alone performed entry of linkage data for patients and RFID tag data.

#### Classification bias

To reduce classification bias, the RFID system determined subject locations independently of the clinical information systems. Off-the-shelf software was utilized to determine contacts. The system was calibrated for accuracy and reliability prior to the start of the study. Initial calibration of the system verified that a radio signal emitted anywhere within the ED footprint would be captured by at least three and typically four receivers. Further verification was completed prior to study initiation to demonstrate that a radio signal received from any location would be accurately mapped to the appropriate zone. No ongoing systematic analysis was performed after the initial setup since hardware and software location and configuration were static. However, the system was utilized during routine, non-research operations, 24/7 during the entire study year, to locate both human and equipment resources in real time. No disparities were noted nor reported by ED staff during this extensive observation. Furthermore, during the twelve-hour study periods, research assistants interacted with the system in real time to locate patients, staff, and equipment. Again, no location disparities were noted. Funding constraints limited our ability to record each instance research assistants interrogated the system for a specific badge location and then found the badge at that location, although research staff were trained to record any operational aberrations.

### Study size

This descriptive study was designed so sufficient data would be available to adequately characterize temporal aspects of contact variability and simulate epidemic spread at seasonally appropriate times of year.

### Statistical Methods

Simple descriptive statistics were utilized to estimate number, duration, and nature of contacts (PP, PS, and SS) during the study period, using SAS v9.3 for Windows 7 Enterprise (SAS Institute, Cary NC). In particular, data were summarized over a shift, and these summary values were further aggregated. Data were highly skewed, thus we present summary percentile values.

Friedman's test [24] along with Tukey's posthoc procedure [25] was used to test if the contact characteristics were the same for PP, PS, and SS using the medians from each shift.

## Results

### Participants

From 730 potential twelve-hour sampling periods, we randomly selected 104 for observation. Usable data were obtained from 81 shifts (78%). The remaining shifts in the sample were excluded due to equipment problems (n = 9; 8%) and insufficient study staffing (due to illness, inclement weather, etc., n = 14; 13%). Over the course of the study year 57,514 distinct patient admissions occurred (Table 1). Of these, 9183 (16%) occurred in the 81 shifts studied. Among these admissions, the research team did not approach 3121 (34%). Of the remaining 6062, 941 (16%) were excluded for patient-related reasons (patient refused assent (38%), patient could not assent (13%), nurses recommended exclusion (15%), imminent discharge (21%), other (13%)). Another 389 (6%) were excluded for technical reasons, leaving 4732 patient-admissions with usable data.

**Table 1:** Characteristics of patients visiting the ED, July 1 2009 to June 30 2010.

	Population from 7/1/2009–6/30/2010		Population in 81 included shifts		Participants in 81 included shifts	
Characteristic	N	% or median (Q1, Q3)	N	% or median (Q1, Q3)	n	% or median (Q1, Q3)
**Total Visits**	57,514	100%	9183	16%	4732	8%
**Age**	57,511	44 (29, 58)	9181	45 (30, 59)	4732	47 (31, 60)
**Female**	32,316	56%	5095	55%	2709	57%
**Black Race**	46,637	81%	7470	81%	3913	83%
**Acuity** 1						
Immediate	955	2%	118	1%	31	1%
Emergent	15,376	27%	2685	30%	1592	34%
Urgent	26,845	48%	4418	49%	2336	50%
Stable	11,992	21%	1659	18%	710	15%
Non-urgent	817	1%	113	1%	32	1%
**ILI syndrome^2^**	732	1%	117	1%	58	1%
**Length of Stay (minutes)**	57,499	314 (213, 444)	9180	355 (245, 507)	4732	381 (270, 549)
**Admitted**	14,405	25%	2766	30%	1686	35%

1 Missing acuity information: 1529 (2.7% of all visits); n = 188 (2.0% in 81 shifts); n = 33 (0.7% of participants). ^2^ILI = influenza-like illness; missing ILI information: 1564 (2.7% of all visits); 203 (2.2% in 81 shifts); 35 (1% of participants).

**Figure 3:**
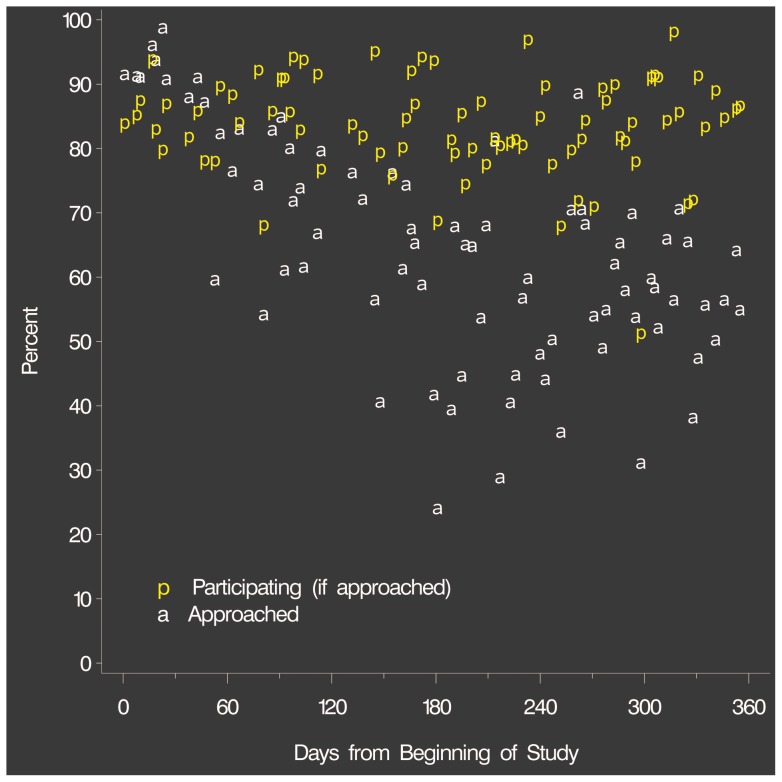
Participation rate and percent of patients approached for each shift versus time, for the study year.

The median percent of patients approached for each shift was 65% and the median percent participating was 85%. As the study progressed, the flux of patients increasingly exceeded the capacity of the research team to approach all patients (Figure 3), although the percentage of patients participating of those approached remained constant. A median of 61 patients /shift were tagged (IQR 46–68).

Demographic and clinical characteristics of the patient admission data are shown in Table 1, summarized for the population over the year, all patients in our 81 sampled shifts, and patient study participants only. There are no clinically meaningful differences between participants and the general population with respect to age, sex, and race. However, participants tended to present with greater acuity. There were parallel increases in length of stay (LOS) and percent admitted to the hospital as the overall patient population narrows to participants.

There were 105 staff eligible, of which 88 (84%) agreed to participate. The median number of staff participants per shift was 28 (IQR 19–36). Although the staffing pattern provided for a maximum of 31 staff per shift, occasional staff meetings, skill seminars, and double coverage caused the number of staff to exceed this maximum in some shifts. As staff are a vulnerable population we did not collect demographic information in order to preserve anonymity and thus maximize participation. Staff participants were observed in shifts numbering between 1 and 77, with a median of 23.

We observed a median of 86 participants per shift (interquartile range (IQR) 73–104). The median of the maximum number of participants in the ED simultaneously was 56 for day and 48 for night shifts.

### Contact Characteristics

Contact characteristics (number per shift and duration) are summarized over 81 shifts (Table 2). Median and IQR values for quantity and duration of contacts within shift and by type of contact (i.e., patient-patient, patient-staff, staff-staff) are shown. Numbers of contact events are described by shift, by participant, and by contact pair. Numbers of contact pairs are described by shift and by participant. Duration of contact is described by total per shift, per contact event, per contact pair, and per participant.

**Table 2:** Summary of contact characteristics per shift among patients and staff in an Emergency Department, over 81 shifts^1^.

	Type of Contact												
	All types			Patient-Patient			Patient-Staff			Staff-Staff			Significant
	Median (1^st^ Quartile, 3^rd^ Quartile) of 81 shifts^2^												Ordering^3^
**Quantity of contacts**													
Contact events^4^/shift	2084	(910, 5706)		459	(278, 610)		261	(119, 660)		1466	(327, 3503)		SS > PP > PS
Contact events/person	22	(9,55)		12	(6, 21)		3	(0, 12)		86	(29, 155)		SS > PP > PS
Contact events/contact pair	2.5	(1.7, 4.1)		2.0	(1.5, 3.0)		2.0	(1.0, 3.0)		6.5	(3.2, 11.0)		SS > PP, PS
Contact pairs^5^/shift	478	(281, 752)		180	(109, 234)		108	(47, 224)		170	(71, 285)		PP > PS
Contacts/person (degree)	9	(5, 17)		5	(3, 8)		2	(1, 5)		13.5	(9, 17)		SS > PP > PS
patient (with staff)							2	(0, 7)					
staff (with patients)							4	(2, 8)					
D**uration of contacts**													
Total hours^6^/shift	426h		(175, 676)	32h		(15, 62)	22h		(8, 47)	272h		(131, 530)	SS > PP, PS
Hours of contact/hour of shift	38.4h		(15.0, 63.1)	2.9h		(1.7, 5.2)	2.0h		(0.8, 4.9)	30.6h		(11.6, 47.9)	SS > PP, PS
Minutes/contact event	3.7m		(1.9, 7.8)	2.9m		( 1.5, 5.0)	2.0m		(0.7, 4.7)	9.3m		(4.2, 16.2)	SS > PP, PS
Minutes/contact pair	5.5m		(0.8, 28.5)	2.6m		( 0.5, 10.7)	2.4m		(0.5, 9.1)	40.2m		(4.8, 186.5)	SS > PP, PS
Minutes/person	106.2m		(27.8, 574.6)	42.1m		(16.8, 87.4)	11.2m		(2.0, 42.3)	974.7m		(211.8, 2378.0)	SS > PP, PS
patient (with staff)							8.9m		(1.6, 32.6)				
staff (with patients)							25.1m		(5.6, 111.5)				

1 There were a total of 185 individuals in 81 shifts that did not make a contact while under surveillance. They are not included in these calculations.

2 The median and quartiles of each shift were calculated and the median of these values are reported. The median of *all types* will not be the sum of the 3 subtype medians.

3 All comparisons across groups types were significant by Friedman's test at p<0.0001, except for contact pairs/shift, which was significant at p = 0.004. Tukey's post hoc procedure was used to determine which groups were different and the ordering.

4 A contact event is defined as any two people being within 1 meter of each other; multiple discontinuous instances between the same two individuals are here counted as multiple contacts.

5 One contact pair is defined as any two people who have at least one instance of being within 1 meter of each other ( =  an edge or link); multiple discontinuous instances are here counted as a single contact.

6 Total hours/shift is the sum of all instances of contact. NB: shift duration ranged from 5 to 12 hours.

We observed a total of 293,181 contact events across all shifts. In the typical observation period, 2084 (median) contact events occurred, with most of type SS. A similar pattern was seen in contact events per participant and per pair. We observed 478 (median) contact pairs per shift, with PP and SS contacts having equally large frequency (180 PP, 170 SS) while PS contacts (108) were less numerous. The number of distinct participants with which any other participant came into contact was largest for SS (13.5) as compared to PP (5) and patient – staff interactions of both types (2). A typical patient participant came into contact with 2 staff participants, while a typical staff participant came into contact with 4 patient participants. In a typical observation period, 426 (median) hours of contact were observed, with SS hours being an order of magnitude greater than PP or PS. Similar patterns were seen with hours of contact per hour of shift. Most contact events were short (<3 minutes for PP and PS, <10 minutes for SS). Total minutes per pair were similarly short for PP and PS (2.6 and 2.4 minutes respectively) but longer by a factor of more than 10 for SS (40.2 minutes). A similar pattern was observed for total duration in contact for participants. A typical patient participant had 8.9 minutes of contact with staff participants, while a typical staff participant had 25.1 minutes of contact with patient participants.

**Figure 4:**
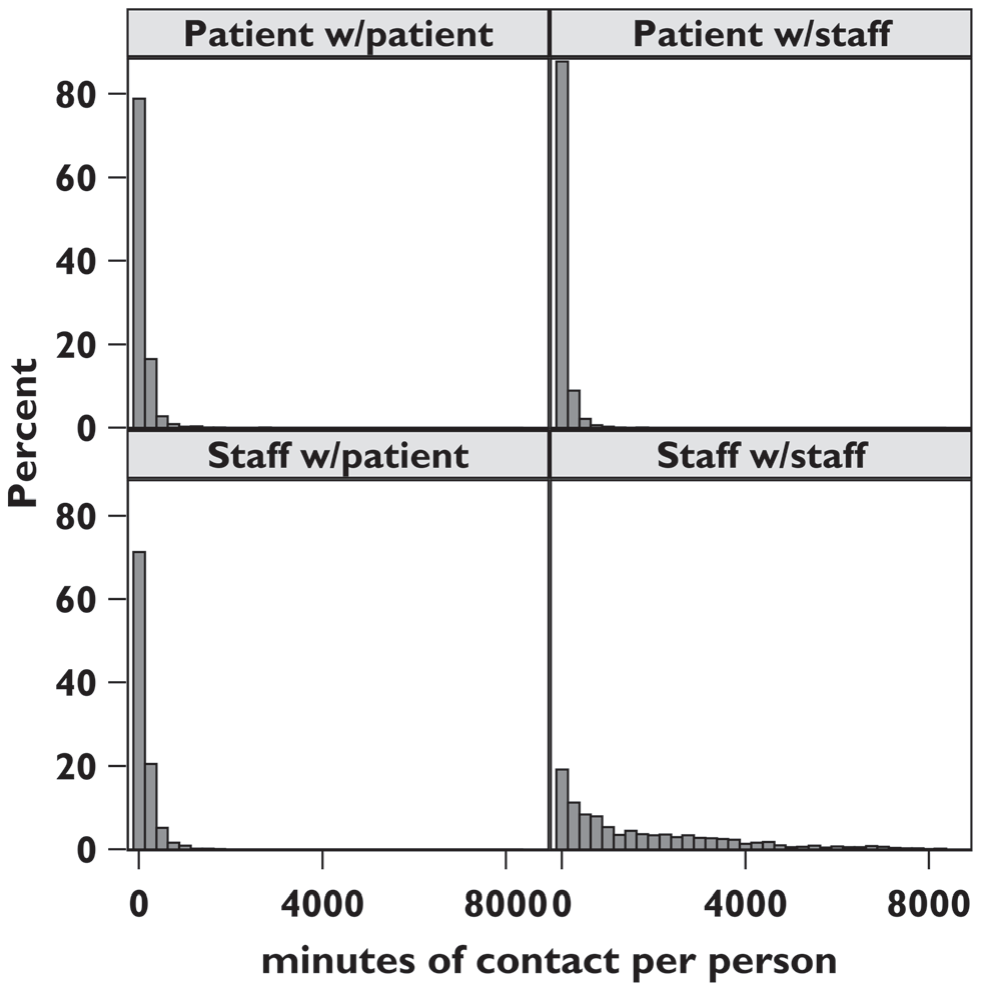
Distributions of the total minutes of contact between participants, by contact type.

**Figure 5:**
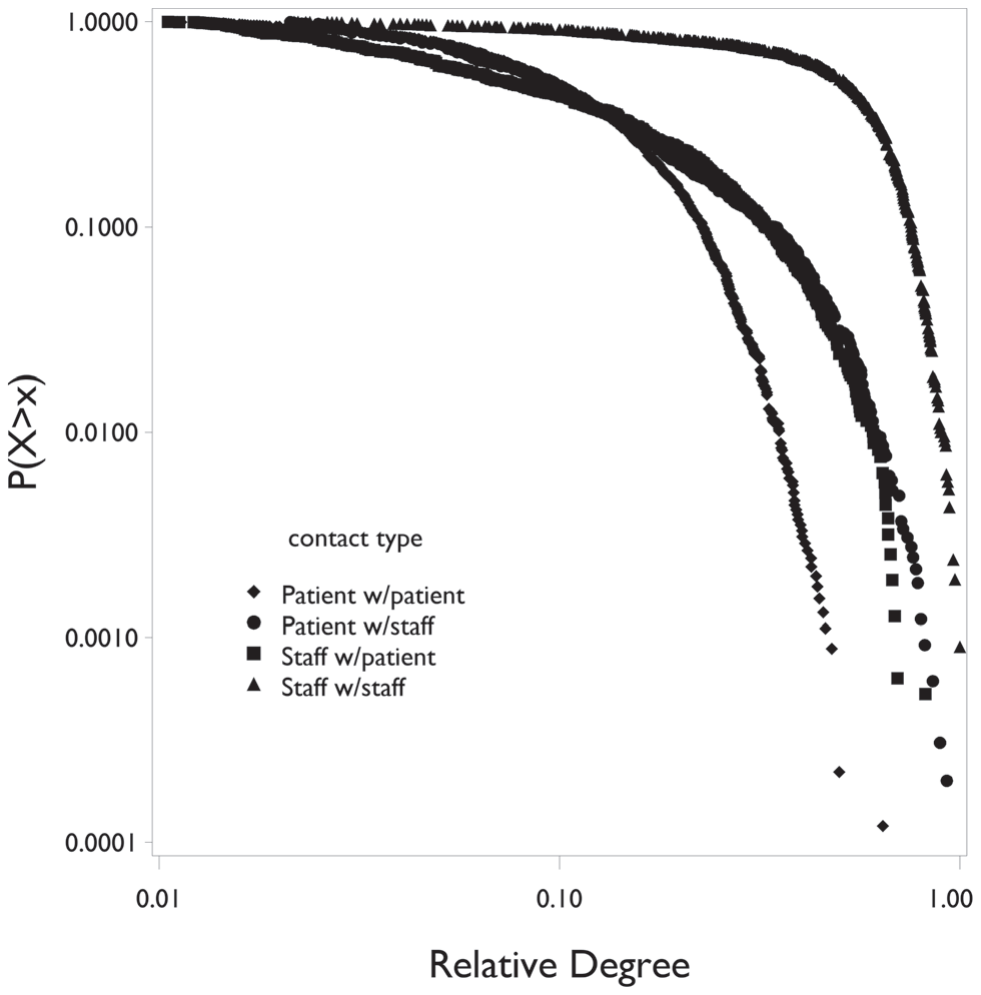
Cumulative distributions of number of contacts per participant (degree) by contact type.

The distributions of the total duration of contacts per participant by contact type (PP, P with S, S with P, and SS) are given in Figure 4. Note that the distribution of total contact duration of staff with other staff is much less right-skewed than for the other contact types. The cumulative distributions of the number of contacts per participant (degree) by contact type are given in Figure 5. Note that these distributions are not consistent with a power law distribution.

## Discussion

### Key results

An off-the-shelf, commercially available, RFID system was used to measure contacts in an ED. The data described here illustrate the complexity of interpersonal interactions in this setting.

We found substantial differences in contact characteristics of the three mixing subgroups. In general, SS interactions were more numerous and longer than PP or PS interactions. Therefore, given a susceptible population of patients and staff, the biological gradient created by individuals in contact favors cross infection among staff. This finding is consistent with previous studies of contacts in the healthcare environment which highlight that applying disease transmission prevention strategies among healthcare workers is an important component of cross infection prevention efforts in healthcare settings. [20]–[23], [26].

### Limitations

The validity of this study for the general healthcare population is limited by several factors.

### Role of Chance

The study period coincided with the novel H1N1 influenza outbreak. The methods of this study made it impossible to know how the novel influenza outbreak or its associated publicity impacts generalizability of our results to the general population of ED's. Informal observations of interpersonal behavior made by research study staff suggest no change in number or duration of interpersonal contacts.

#### Sources of Bias

Despite our countermeasures, several types of bias were present in the study, which might result in biased estimates of number and duration of contacts among all mixing groups.

Several factors led to the presence of selection bias. First, the study was performed in a busy, urban ED with unique facility footprint, staffing pattern, and patient demographics. Therefore these results may not be applicable to all ED's or even to other similar, non-ED healthcare environments. Study criteria excluded visitors and non-ED based hospital staff present in the ED (e.g., cleaning staff, hospital chaplains) as well as prehospital personnel. Moreover, only 88 staff participated, although 105 staff were employed during the study year. IRB approval was contingent on anonymization of staff beyond job category (physician, nurse, other patient care, administrative). Therefore, number and total duration of staff contacts are underestimated.

Second, over time we observed fewer RFID signals from staff participant tags. While it is reasonable to conclude that there was a decline in staff participation over the course of the year, there is also high probability that some (and perhaps most) of the decline could be attributed to battery failure in the permanent tags. The battery half-life was one year, and these badges were activated six months prior to the commencement of our year of official study observation in order to test and calibrate the system. Indeed, the fall-off in staff participation is consistent with exponential failure time with half-life of one year. A spurious system setting prevented the receipt of planned alerts regarding weakening batteries. After the conclusion of the study period, we found a substantial number of staff tags with dead batteries. Thus the fall-off in staff participation is likely due to battery failure rather than staff selecting out of the study. Regardless of the reason, the effect of this decline is underestimation of the number and duration of contacts.

Lastly, despite utilizing randomly selected observation periods and waiver of all elements of informed consent, patient participants tended towards higher acuity triage score, higher likelihood of hospital admission, and longer ED LOS than the ED population at large.

The study may also be affected by measurement bias. Study staffing was inadequate to provide tagging of patients at the instant of ED arrival. Thus actual LOS was longer than studied LOS, so number, degree, and duration of contacts have likely been underestimated. Moreover we made a protocol decision not to tag patients who were awaiting discharge. In this case, the number and duration of contacts have also likely been underestimated.

On the other hand, we assumed that discrete contact events occurred when the adjacency matrix elements of the TRAO for subjects i and j along the time dimension were two strings of 1′s separated by as few as three 0′s. Such an occurrence could reflect, for instance, a staff member seeing a patient in an exam room (series of 1′s), stepping out of the room for as little as three seconds (0, 0, 0), then returning to the room (series of 1′s). Since we could not separate these very real possibilities from those due to poor signal quality, we elected to leave these as separate events. In this case, we may have overestimated the number of contact events while underestimating event duration.

Another place where measurement bias may have occurred was in the waiting room. Our focus on getting greater separation between patient exam rooms came at the expense of less separation in the waiting room, a large (∼2500 square foot) open square-shaped area that was divided for system purposes into two zones. All participants that the system located to one waiting room zone were counted as in contact. Given the hard and soft architectural features of this space, it is highly likely that individuals colocated within the space would have a high probability of being in close personal contact. However, we could have overestimated the number and duration of contacts for patients in one waiting room zone that were actually more than 1 m apart, but we also could have underestimated the number and duration of contacts for patients in two zones that in reality were seated next to each other. The waiting room population is very dynamic. There are a number of factors that contribute to this. The waiting room lies between the main entrance for walk-in patients and the registration area. Thus upon first entry patients and/or their accompanying visitors must walk through the thick of other patients to sign into the system. Second, the front-end process of care requires patients to make multiple trips to and from the waiting room: sign-in, triage, hospital registration, care initiation, emergency radiology, and, in some cases, to see a mid-level provider. Also, to enhance patient satisfaction with the ED visit experience, the waiting room contains many diversions to address patient comfort, for example, a coffee station, telephones, reading materials, vending machines, restrooms, and, during the time of the study, a designated smoking area. All of these factors contribute to high mobility of patients in the waiting room during their visit, resulting in more brief contacts than might initially be expected. In the case of a highly transmissible virus, such patterns of contacts may be sufficient for cross infection. However, for a less transmissible virus, many brief contacts may not generate sufficient exposure to the index case that would result in cross infection.

Several factors contributed to classification bias. Software requirements mandated division of the ED footprint into mutually exclusive zones, for which any two individuals simultaneously in a zone would likely (but not certainly) be in contact with one another. The net effect of this procedure should result in neither overestimation nor underestimation of study results.

Challenges were encountered among staff compliance with badges. Hospital staff members typically work in designated areas, and thus perceive themselves to be in constant visual contact with their team members. Therefore, to the staff, benefits to be obtained by tracking potential cross infection exposures are outweighed by costs – for instance, annoyance due to dropping of tag when affixed to pocket, tangling with stethoscope when carried on lanyards around the neck, or interfering with hospital staff ID tags. We attempted to improve staff compliance by issuing permanent tags for staff to wear and providing periodic education sessions.

Staff did not have to activate their tags nor record their use, therefore we had a completely passive inclusion system for staff but not for patients. For staff, we considered the shift start time as the time that the tag was first located outside staff only areas or the observation period begin time, whichever occurred last. Similarly the shift end time was the time that the tag was last located outside staff only areas, or the observation period end time, whichever occurred first. This was done in order to account for tags that might be stored in a locker or left on a sweater draped over a desk chair when the staff member was not working.

### Interpretation

Most importantly the number and duration of staff-staff contacts demonstrate the dangers of ill or infectious staff members at work. This finding is consistent with simulations conducted by others [22], [23]. If transmission is related to a biological gradient of exposure as defined by magnitude and duration of contacts, then pathogens transmissible by droplets appear to have a higher likelihood of cross infection from working contagious staff (i.e. “presentees”) to susceptible peers. In the case of annual influenza outbreaks, this finding underscores the importance of vaccination of healthcare employees assigned to the ED in order to prevent health care service interruption due to widespread staffing illness (i.e. absentees) since infectious humans may be shedding virus hours or days prior to developing symptoms. In the case of novel infectious diseases transmissible by droplets, it also underscores the risk for staff cross infection once one staff member has been infected. Healthcare systems might consider keeping staff in droplet precautions even when not with symptomatic patients, as well as other efforts to reduce the likelihood of cross infection among staff in general. ED staff tend to look at their patients as high risk, while viewing other staff as “safe” unless symptomatic. However, implementation of infection-control measures is more difficult in the ED than in other hospital areas, since patients' conditions have not been identified upon arrival. ED staff are at higher risk for cross infection than personnel in other hospital areas [27].

Interestingly, the number of contacts per participant is not consistent with a power law distribution, indicating that our networks are not scale-free. This finding was unexpected *a priori*, since this property has not been found for other common types of networks, although it has been found by Gundlapalli et al. in a study of contacts in a pediatric ED [20]. As Gundlapalli and colleagues noted, patients and staff in particular do not associate in a manner that would be appropriate for a preferential attachment model that would give rise to a power law distribution – newly arriving patients are assigned to staff as staff discharge their current patients. Importantly, mathematical models of cross infection in the ED that assume contact networks that are scale-free in nature may not describe the cross infection process correctly.

### Generalizability

Five other studies are directly comparable to ours. Polgreen and colleagues report results of shadow observations of staff over 40-hour observation periods during one year throughout a large, academic, rural hospital [26]. The authors found 71% of staff contact events were with other staff. Our results are compatible with this observation. Isella et al. report the results of one continuous week of RFID determination of contacts between staff, patients, and visitors in a general pediatric hospital in Rome, Italy [22]. There are too many substantial differences in study design (pediatric vs adult populations, observation continuous over one week vs 12 hour shifts throughout one year, general hospital ward vs emergency department) to compare their findings to ours. Lucet and colleagues implemented an RFID system in two French hospital wards (one infectious disease, one pulmonology) over a three-month period in order to determine contacts between staff and patients under respiratory precautions due to tuberculosis [21]. Implicit in these precautions is severe curtailment of the possibility of PP contacts, precluding direct comparison with our results.

Most recently Hornbeck and colleagues report on the use of a mote-based sensor system to characterize the locations of staff in a 20-bed MICU [23]. Data were collected for only seven days, and reported for only two days. Only staff were given wearable badges, whereas fixed beacons were placed in patient rooms and in other commonly shared patient care areas (e.g., hallways, nurses' station). No patient-specific data were collected. There were 16 staff present, on average, in each shift, in comparison to our observation of a median of 28 staff per shift. In the four shifts reported, the typical staff had approximately 50 (median) contacts, with SP contacts less than SS contacts and for both day and night shifts. Contacts were short, typically less than 1 minute (median), for both SP and SS contacts and for both day and night shifts. These were much fewer and much shorter than the contacts we observed. Other than the difference in the purpose of the unit, another factor that might account for the differences between our observations and theirs might be the area of the unit footprint, which we could not determine.

In the study with the most comparable setting and methods, Gundlapalli and colleagues report data on contacts between 1261 patients and 87 staff in a pediatric hospital ED collected over the course of a randomly chosen month [20]. They constructed networks from existing clinical informatics resources, notable a proprietary patient flow management system as well as a locator system for which staff were given IR badges to wear. In this case, locations of staff were zoned, then merged with the patient flow system data to create a dataset describing PS interactions. The system did not cover the waiting room, in contrast to ours. Each staff had contact, on average, with 6 patients, while each patient had contact on average with 3 staff. They also delimited these data for one day, and the resulting network described interactions among 21 staff and 40 patients. The average contact duration per pair was 20.16 seconds, and the average number of contacts per participant was 4.86. For both analyses, SS and PP interactions were not considered. The degree distribution was not consistent with a power law distribution, as we also observed. Coupled with our observation, there may be some other forces at work here, which may play out in the cross infection process. Certainly, as these authors note, there is not a preferential attachment model working for staff-patient assignments, as at least the initial contacts have more to do with the length of the queue of patients for which the staff are already caring.

Although the two hospital-based RFID studies are not directly comparable to ours, both studies report issues with participation and with the system similar to our experience. In the Italian study there was excellent participation (96.6%), but data from approximately half of the patients (39/76) and visitors (30/61) had to be excluded for technical reasons [22]. Among 79 days of observation in the French study, less than half could be used in the analysis due to reasons similar to ours (i.e. failure of staff to carry tag, technical issues) [21].

RFID systems and other remote location sensor technologies will find greater applications in health care systems in the future, with increasing technical capabilities. We have demonstrated that social contacts in the ED can be quantified over long periods. This paper has barely tapped this rich data resource. In future papers we will explore the dynamics of the social networks we have characterized by relating our contact metrics with staff and patient characteristics as well as with the stages of patient care. Our findings will also inform mathematical models and simulation studies to determine the potential risks of cross infection and the likelihood of the infection spreading to the rest of the hospital through an admitted patient cross infected in the ED. Considering patient and staff interactions in the frame of a network opens up possibilities for major improvements not only in infection control, but also in facilities design and in work- and patient-flow management.
